# Exploring resilience in critical care nursing: a qualitative inquiry into continuous adaptation, collaborative unity, and emotional balance

**DOI:** 10.1186/s12912-025-02844-0

**Published:** 2025-03-03

**Authors:** Eman Arafa Hassan, Shimmaa Mohamed Elsayed

**Affiliations:** 1 Critical Care and Emergency Nursing, Critical Care and Emergency Nursing Department, Faculty of Nursing, Alexandria University, Alexandria, Egypt; 2 Critical Care and Emergency Nursing, Critical Care and Emergency Nursing Department Faculty of Nursing, Damanhour University, El Beheira, Egypt

**Keywords:** Critical care, Emotional well-being, Nurses, Resilience, Teamwork

## Abstract

**Background:**

Resilience is essential for nurses in critical care, where they face high-stakes situations requiring continuous adaptation, collaborative unity, and emotional balance. This study aimed to investigate resilience in critical care nursing, focusing on how nurses adapt to challenges, the role of teamwork in fostering resilience, and strategies for maintaining emotional balance.

**Objective:**

The objective of this study was to explore resilience among critical care nurses, emphasizing their adaptation to challenges, the influence of collaborative practices, and methods for sustaining emotional well-being in intensive care units.

**Methods:**

A qualitative study with a thematic analysis approach was used in this study. The study was conducted in five intensive care units across two hospitals in Egypt. The purposeful sampling approach includes 17 critical care nurses with diverse experiences. Data was collected through in-depth semi-structured interviews using an interview guide focusing on challenges, adaptation strategies, collaborative practices, and coping mechanisms.

**Results:**

Themes emerge, depicting resilience as a dynamic process encompassing continuous adaptation, learning, collaborative unity, emotional balance, self-care, and reflection on experiences. Nurses emphasize the importance of teamwork, interprofessional collaboration, and managing emotional complexities. Resilience is portrayed as a collective force within the critical care team, balancing compassion and clinical precision.

**Conclusion:**

Critical care nurses demonstrate resilience as a multifaceted and dynamic process. The study provides insights into the collaborative strategies employed and the emotional aspects of resilience. Acknowledging vulnerabilities and prioritizing self-care are integral to sustaining resilience.

**Supplementary Information:**

The online version contains supplementary material available at 10.1186/s12912-025-02844-0.

## Introduction

Resilience in nursing refers to the ability of nurses to cope with and adapt to the challenges and stressors they encounter in their work. It involves the capacity to recover from difficult situations, maintain a positive outlook, and continue to provide high-quality care to patients despite the demands of the job [1, 2]. Resilience in nursing is essential for preventing burnout and sustaining a fulfilling and effective nursing practice [3].

Resilience is a fundamental trait that enables individuals to bounce back from adversity and maintain their well-being [4]. In the demanding field of critical care nursing, resilience plays an important role in ensuring the physical and mental health of nurses. These healthcare professionals face numerous challenges daily, including high patient acuity, long working hours, emotional stress, and ethical dilemmas [5].

The critical care demanding field requires practitioners to navigate a complex interplay of challenges, ranging from high-stakes medical interventions to emotionally charged patient interactions [6]. Resilience in this context encompasses the ability to continuously adapt to dynamic challenges, maintain collaborative unity within the healthcare team, and achieve emotional balance. Despite the acknowledged significance of resilience, there remains a dearth of in-depth qualitative exploration into the nuanced experiences of critical care nurses [7, 8].

Studies on resilience in critical care nursing have shown that resilience is a crucial trait for nurses working in high-stress environments [9, 10]. Research has highlighted the importance of resilience in helping critical care nurses cope with the emotional and psychological demands of their work [8]. Studies have also indicated that resilient nurses are better able to adapt to challenging situations, manage stress, and maintain a positive outlook in the face of adversity [9, 10].

Resilience in nursing is not only vital for the well-being of nurses but also profoundly impacts patient care. Research has shown that resilient nurses are better equipped to maintain focus and composure in high-pressure situations, directly contributing to patient safety and minimizing the risk of medical errors [11]. Moreover, their ability to adapt to dynamic clinical environments can enhance patient access to timely and effective care, as resilient nurses are more likely to maintain high performance levels under stress. This adaptability can also improve patient satisfaction, as patients often perceive a higher quality of care when healthcare professionals exhibit empathy, professionalism, and calmness during critical situations [12].

Resilience in critical care nursing has been associated with lower levels of burnout, compassion fatigue, and emotional exhaustion [13]. It has also been linked to higher job satisfaction and overall well-being [14]. Research has explored various factors that contribute to resilience in critical care nurses, including social support, coping strategies, self-care practices, and organizational support [7, 15].

Understanding the dynamics of resilience in critical care nursing holds profound implications for fostering a resilient workforce, ultimately enhancing patient care, and shaping the future of healthcare practice [8]. The significance of this study is underscored by the growing recognition of the impact of nursing resilience on patient outcomes, healthcare system efficiency, and the overall quality of patient care. Previous research has highlighted the positive correlation between nursing resilience and job satisfaction, as well as its potential to mitigate burnout and improve retention rates in high-stress environments. However, the specific components of resilience that contribute to these outcomes in critical care nursing remain underexplored [16, 17].

Despite the growing body of literature on nursing resilience, significant gaps remain in understanding its broader implications, particularly in critical care settings. Existing studies often focus on individual factors contributing to resilience but less frequently address how these factors interact with the work environment to influence patient care outcomes such as safety, access, and satisfaction. Additionally, while research highlights the protective role of resilience against burnout and emotional exhaustion, there is limited exploration of how resilience can mitigate systemic challenges in healthcare, such as staffing shortages and workflow inefficiencies [18, 19]. By addressing these gaps, this study seeks to provide a more holistic understanding of resilience, ultimately contributing to the development of targeted interventions that benefit both nurses and patients.

This study seeks to address this gap by delving into the multifaceted nature of resilience, aiming to uncover the intricacies that contribute to the adaptive capacity and overall well-being of these frontline healthcare professionals. By offering valuable insights that can inform both clinical practice and policy development within the healthcare sector, this study has the potential to shape the future of critical care nursing and enhance patient outcomes [20, 21].

## Methods

### Aim

This study aims to explore resilience in critical care nursing. Specifically, the study aims to explore how these nurses continuously adapt to the challenges inherent in critical care settings, the role of collaborative unity in fostering resilience within nursing teams, the strategies employed to maintain emotional balance in high-stakes situations, and the identification of factors influencing resilience.

### Research design

This study employs a qualitative research design with a focus on thematic analysis. Thematic analysis is chosen as it allows for the identification and exploration of patterns, themes, and variations within the rich narratives provided by critical care nurses. It is particularly suitable for uncovering the nuanced experiences and perspectives related to resilience in the dynamic context of an intensive care unit (ICU) [22, 23]. This study was reported according to consolidated criteria for reporting qualitative research (COREQ) checklist [24] as a Supplementary file 1.

### Settings and context

The study was conducted in five ICUs across two different hospitals in Egypt. Specifically, these included three general ICUs, two neurological ICUs, and a medical ICU. The selection of these diverse ICUs aimed to capture a comprehensive understanding of resilience in critical care nursing across various specialized units, each presenting unique challenges and dynamics.

The inclusion of both general and specialized ICUs enhances the richness and breadth of the data, allowing for a more nuanced exploration of continuous adaptation, collaborative unity, and emotional balance among critical care nurses in different clinical settings. This geographic and unit diversity contributes to the generalizability of findings and provides a holistic perspective on the experiences of resilience in the dynamic context of Egyptian critical care nursing.

Furthermore, the choice of multiple hospitals adds another layer of variability, considering that hospital cultures and practices may influence the experiences of resilience among critical care nurses. This multisite approach strengthens the robustness of the study and allows for a more comprehensive understanding of the phenomena under investigation.

### Participants

A purposeful sampling approach was utilized, involving the inclusion of 17 critical care nurses. Out of 25 nurses approached, 17 agreed to participate, while 8 declined due to time constraints or personal reasons. Sampling continued until data saturation was achieved, ensuring a comprehensive understanding of the participants' experiences. After seeking permission from unit managers and introducing the study to them, the researchers asked for their support in identifying potential participants among their nursing staff. Participants were selected based on their track record of demonstrating resilience in their professional roles, which was evidenced by facing challenging situations in critical care, adapting to changes, and maintaining a positive attitude despite adversity. Following the unit managers' introductions, the researchers directly contacted potential participants in person within their respective units to explain the study in detail and invite them to participate. This direct contact allowed for immediate clarification of any questions and facilitated the informed consent process.

Another inclusion criterion was diverse experience levels, including participants with varying years of experience and different critical care settings. This diversity provides a range of perspectives and contributes to a more comprehensive understanding of how resilience evolves over the course of a nursing career. Nurses were excluded if they had less than one year of experience in critical care settings or if they did not demonstrate a track record of resilience in their professional roles. Nurses with less than one year of experience in critical care settings were excluded because participants with limited professional experience may not have encountered a broad range of challenges and may not have had the opportunity to develop and demonstrate resilience in various situations. Furthermore, the exclusion of those without a demonstrated track record of resilience ensured that the study focused on nurses who had actively navigated challenging situations and exhibited adaptive coping mechanisms.

### Tool

The data collection tool employed for this study was an interview guide featuring semi-structured questions, meticulously developed by the researchers. The questions aimed to elicit rich and detailed responses from critical care nurses regarding their experiences with resilience. The initial set of questions encompassed various dimensions of resilience in critical care nursing. These questions were designed to facilitate in-depth exploration of the participants' experiences, allowing for the emergence of themes and patterns related to resilience in critical care nursing. Since the interview guide was developed exclusively for this study and has not been published elsewhere, an English language version of the interview guide is provided as a Supplementary file 2.

### Data collection

After obtaining ethical approval, two researchers collaborated with unit managers and seek permission to introduce the study to potential participants among the critical care nursing staff. Critical care nurses were invited to participate in the study. The researchers administer informed consent procedures, clearly outlining the study's objectives, the voluntary nature of participation, and the confidentiality of data. In addition, they encouraged participants to ask questions and express any concerns before obtaining their consent.

In-depth semi-structured interviews were conducted using the pre-developed interview guide to allow nurses to share their experiences, challenges, and strategies related to resilience. With participants' consent, the researchers recorded the interviews using audio record to capture detailed responses accurately. The researchers ensured that participants were comfortable with the recording process and reassured them of confidentiality. Field notes were taken during and after each interview to document observations, reflections, and any contextual information that may contribute to the analysis.

Interviews continued until data saturation was achieved, ensuring a comprehensive understanding of the participants' experiences. No repeat interviews were conducted as data saturation was achieved within the initial interviews. Transcripts were not returned to participants for comment or correction; however, member checking was conducted by sharing preliminary findings with participants to validate the emerging themes.

Interviews took place in nurses’ resting rooms within the ICUs to ensure confidentiality. No non-participants were present during the interviews to ensure privacy and confidentiality. Each interview took from 30 to 45 min. This study was conducted in Egypt, and data were collected in the Arabic language to ensure a nuanced exploration of the experiences of critical care nurses within their cultural context.

### Data analysis

The data analysis process adhered to established principles of thematic analysis, allowing for a systematic and rigorous exploration of participants' responses [22, 23]. The researchers transcribed the recorded interviews verbatim, ensuring a faithful representation of the nuanced content within participants' narratives. This approach preserves the depth and intricacies of the data, aligning with the principles of qualitative inquiry.

The two researchers independently coded the data to ensure reliability and consistency in the analysis. A coding tree was developed to organize the data, with initial codes grouped into broader themes and subthemes. The coding tree included categories such as continuous adaptation, collaborative unity, and emotional balance, which were further refined during the analysis.

Thematic analysis, as outlined by Braun and Clarke (2006) [22], was employed to unravel the patterns, themes, and subthemes embedded in the interview transcripts. Initial coding commenced through a meticulous and iterative process of closely reading the data, generating preliminary codes, and actively searching for recurrent themes. This inductive approach ensured a grounded and data-driven identification of patterns within the critical care nurses' experiences of resilience.

The identified themes underwent a rigorous analytical review, involving iterative cycles of refinement and organization. This process aimed to enhance the coherence and internal consistency of the emerging themes, aligning them with the study's overarching focus on continuous adaptation, collaborative unity, and emotional balance. The final thematic framework was then systematically organized into a coherent narrative, capturing the essence of critical care nurses' experiences and providing a robust foundation for interpreting the study's findings.

The data were analyzed manually without the use of qualitative data analysis software. The researchers conducted the coding, theme development, and organization of the data through a systematic and iterative process, ensuring rigor and transparency in the analysis. Certified translators, fluent in both Arabic and English, were engaged in the translation process. These translators possessed a deep understanding of the healthcare context and cultural intricacies pertinent to the study. To validate the accuracy of the translations, a back-translation procedure was implemented by an independent translator who did not have access to the original Arabic data.

### Rigor and trustworthiness

Data collection for this study was conducted collaboratively by two researchers to ensure thoroughness and multiple perspectives in the interpretation of critical care nurses' experiences. To enhance the credibility and confirmability of the findings, member checking was implemented by sharing preliminary findings with participating nurses. This iterative process involved seeking their feedback and insights, allowing for a validation of the emerging themes and interpretations.

To further strengthen the study's rigor, a third researcher, recognized as an expert in qualitative research, played a crucial role in peer debriefing and reflexivity. This external expert engaged in critical discussions with the primary researchers, providing valuable insights and alternative viewpoints. These discussions aimed to minimize potential biases and enrich the analytical process, contributing to the overall trustworthiness of the study.

Additionally, an audit trail was diligently maintained to document the decisions made throughout the data analysis process. This systematic record-keeping ensures transparency and accountability, allowing for an audit of the analytical journey. The audit trail serves as a valuable resource for external reviewers to evaluate the study's methodological rigor and the logical progression of analytical decisions.

### Reflexivity and positionality

Throughout the research process, the researchers maintained a critical awareness of their own backgrounds, experiences, and potential biases. Author 1, an assistant professor at Alexandria University with a PhD in Critical Care and Emergency Nursing, and Author 2, a lecturer at Damanhour University with a PhD in the same field, conducted the interviews. Both researchers were female, which may have influenced participants' openness during interviews, particularly when discussing sensitive or emotionally charged topics. Both authors have extensive experience as critical care nurses and completed advanced training in qualitative research methods, including interview techniques and thematic analysis. They also have previous qualitative and mixed research publications.

The researchers acknowledged that their shared professional background provided valuable insights into the context of critical care nursing, informing the development of the interview guide and facilitating a deeper understanding of the participants' experiences. The study aimed to explore resilience in critical care nursing, with a specific focus on continuous adaptation, collaborative unity, and emotional balance. This interest in resilience may have influenced the researchers' focus during data collection and analysis, particularly in identifying themes related to coping mechanisms and teamwork. However, the researchers also recognized that their shared experiences could potentially lead to assumptions or biases in the interpretation of the data. For example, as critical care nurses, they were particularly attuned to the emotional and psychological challenges faced by nurses in high-stress environments, which may have shaped the framing of interview questions and the interpretation of responses.

To mitigate this risk and enhance the trustworthiness of the study, the researchers engaged in ongoing discussions, reflexive journaling, and peer debriefing with a third researcher who had expertise in qualitative research but no direct experience in critical care nursing. The third researcher provided an external perspective during peer debriefing sessions, challenging the researchers' interpretations and ensuring a balanced analysis.

Participants were informed that the study aimed to explore resilience in critical care nursing, with a focus on continuous adaptation, collaborative unity, and emotional balance. They were assured of the researchers' commitment to maintaining confidentiality and objectivity. No prior relationship existed between the researchers and participants, ensuring that the data collection process remained unbiased and focused on the participants' perspectives.

### Ethical considerations

The researchers obtain ethical approval from relevant review boards before initiating any data collection activities. The researchers elaborated on the study's purpose and obtained informed consent from all participants. The anonymity and confidentiality of participants were strictly maintained.

## Results

Interviews were conducted with 17 critical care nurses until saturation was achieved. The median age of the nurses was 42 (ranging from 24 to 57). They had a median of 16 years of experience in critical care settings (ranging from 2 to 35 years). Among the nurses, three had previous experience in trauma ICU before working in general ICUs. Two nurses had previous experience in a stroke unit before transitioning to the medical ICU. Six nurses had their entire experience in neurological ICUs, while three nurses had their total experience in general ICUs. Lastly, three nurses had their total experience working in a medical ICU.

Our thematic analysis revealed five profound themes that offer insights into the dynamic nature of resilience among ICU nurses. We explored the intricate fabric of resilience within the context of critical care nursing, as illustrated in Fig. 1.

### Resilience as a dynamic process

The ever-shifting landscape of the ICU demands constant adaptation from its nurses. Resilience is not a static attribute, but a dynamic process of navigating the unpredictable. Nurses embrace a mindset of continuous learning, viewing challenges as stepping stones to evolve their skills and knowledge for the betterment of patient care. They adapt to the ever-changing environment, recognizing that staying flexible and proactive is key to success.

#### Continuous adaptation

The ICU environment, with its constant flux, demands a resilient response. Nurses dynamically adapt to the ever-changing and unpredictable nature of the ICU, recognizing that resilience is not a static trait but an active process.

“The ICU is like a rollercoaster ride – every day, every moment is different. You have to be able to roll with the punches, adapt to the changes, and always be ready for the next challenge. Resilience is not about being perfect; it is about being able to keep going, even when things are tough.”—ICU Nurse 12.

#### Learning and growth

Resilience unfolds as an ongoing journey of personal and professional development. Nurses view challenges as stepping stones, committing to a continuous process of learning, adaptation, and evolution for the betterment of patient care.

“I have learned so much since I started working in the ICU. Every patient, every situation, is a learning experience. I have learned to trust my instincts, to think on my feet, and to never give up. Resilience is about constantly learning and growing, even when you make mistakes.”—ICU Nurse 8.

### Collaborative resilience

Within the ICU, individual strengths blend to form a powerful network of resilience. Teamwork becomes fundamental, where challenges are navigated collectively, burdens shared, and victories celebrated together. Nurses trust and respect each other's expertise, fostering a strong sense of unity that transcends individual capabilities. This collaborative spirit extends beyond the nursing team, embracing interprofessional collaboration with doctors, therapists, and specialists to build a resilient ecosystem of care.

#### Team unity

Collaborative resilience emerges as a cohesive force within the ICU team. Individual strengths amalgamate into a formidable unit, where resilience is not only about personal endurance but a collective strength that navigates challenges, shares burdens, and emerges stronger together.

“The ICU is a team sport. We all have to work together to provide the best possible care for our patients. We share the load, we support each other, and we celebrate our successes together. Resilience is about knowing that you're not alone, that you have a team of people behind you every step of the way.”—ICU Nurse 1.

#### Interprofessional collaboration

Resilience flourishes within the collaborative ecosystem of healthcare professionals in the ICU. Nurses and specialists contribute as vital components, fostering mutual trust, shared responsibility, and unwavering support to fortify the team's resilience, especially in the face of demanding cases.

“In the ICU, we have to work with a lot of different people – doctors, therapists, specialists. But we are all united by a common goal: to provide the best possible care for our patients. Resilience is about respecting each other's expertise and working together to achieve that goal.”—ICU Nurse 11.

### Emotional balance

Balancing deep empathy with clinical precision is a cornerstone of resilience in critical care. Nurses navigate the emotional complexities of the ICU by seamlessly integrating compassion into their clinical skills. They maintain a delicate equilibrium between emotional connection with patients and the clinical detachment necessary for making crucial decisions. This emotional balance allows them to provide patient-centered care with both strength and sensitivity.

#### Compassion and clinical precision

Navigating the emotional complexities of critical care, nurses strike a delicate balance between deep empathy and clinical precision. Resilience is defined by the fusion of compassion and clinical skills, enabling them to provide patient-centered care with strength and grace.

“We have to be compassionate and empathetic, but we also have to be able to make tough decisions. It's a delicate balance, but it's essential for providing the best possible care. Resilience is about finding that balance and being able to hold both compassion and clinical precision in your heart.”—ICU Nurse 5.

#### Coping with challenges

Resilience involves managing not just patients but also the emotional toll on nurses themselves. Coping with challenges is an integral aspect, requiring inner strength to persevere through emotional stressors, ensuring continued dedication to their demanding roles.

“The ICU is a lot to handle emotionally. We see a lot of suffering, and we lose patients sometimes. It's tough, but we have to find ways to cope. Resilience is about being able to bounce back from setbacks, to find strength in the face of adversity.”—ICU Nurse 9.

### Self-care and well-being

Acknowledging vulnerability becomes a key aspect of resilience in the ICU. Nurses understand that in this demanding environment, embracing their vulnerabilities is not a sign of weakness, but a testament to their humanity and the importance of self-awareness. They prioritize self-care, recognizing it not as a luxury but as a professional responsibility. By taking intentional steps to nurture their well-being, nurses prevent burnout and sustain their resilience to provide consistent, high-quality care.

#### Acknowledging vulnerability

Recognizing vulnerabilities becomes a cornerstone of resilience in the ICU. Nurses acknowledge that in the demanding environment, embracing vulnerability is not a sign of weakness but a testament to their humanity, reinforcing the importance of self-awareness.

“We are all human, and we all have our limits. It's okay to ask for help when you need it. In fact, it's essential for resilience. Resilience is about acknowledging your vulnerabilities and taking care of yourself.”—ICU Nurse 15.

#### Prioritizing self-care

Prioritizing self-care is positioned not as a luxury but as a professional responsibility. Nurses understand the necessity of intentional steps to nurture their well-being, recognizing that self-care is essential for sustaining resilience and preventing burnout.

“I used to think that self-care was a luxury, but now I know it's a necessity. If I'm not taking care of myself, I'm not going to be able to take care of my patients. Resilience is about making time for yourself, even when you're busy.”—ICU Nurse 6.

### Reflection on experiences

Resilience flourishes in the spaces between challenges. Nurses find strength not only in overcoming obstacles, but also in acknowledging and celebrating victories, no matter how small. Reflecting on these successes fuels their motivation and offers a renewed sense of hope. Additionally, they embrace setbacks as opportunities for learning and refinement. By actively analyzing difficult cases and turning them into learning experiences, nurses build resilience and improve their skills for future challenges.

#### Celebrating victories

Resilience manifests in the spaces between challenges, where nurses acknowledge and celebrate victories, no matter how small. Reflecting on these successes becomes a guiding compass, offering hope and renewed strength.

“Every small victory in the ICU is a big deal. It's important to take the time to celebrate our successes, no matter how small. Resilience is about finding joy in the little things and savoring the moments of triumph.”—ICU Nurse 12.

#### Learning from setbacks

Every setback in the ICU becomes an opportunity for reflection and refinement. Resilience-building takes place as nurses actively learn from difficult cases, turning setbacks not with regret but with determination to improve.

“Mistakes happen in the ICU, but it's important to learn from them and move on. Resilience is about not giving up on yourself, even when you make mistakes. It's about using setbacks as opportunities to grow.”—ICU Nurse 7.

## Discussion

Our exploration into the resilience of critical care nurses has unearthed rich narratives, offering profound insights into the dynamic processes at play within the ICU setting. The thematic analysis illuminated several key themes and subthemes that collectively paint a comprehensive picture of resilience in this demanding healthcare environment.

As regards continuous adaptation, the ICU is characterized by constant unpredictability and challenges [25]. The nurses' accounts underscore the imperative of continuous adaptation. The ability to swiftly rewrite strategies in response to the ever-changing landscape is not only a testament to individual resilience but also a reflection of the dynamic interplay between nurses and their demanding work environment [26]. The symbiotic relationship between nurses and the ICU environment becomes evident, as the nurses draw on their experiences and expertise to not only confront challenges but also influence and refine the very nature of the environment they work in [27].

Nurses described their role as a perpetual learning journey, where each shift introduces new challenges and opportunities for growth [28]. This aligns with the subtheme of “Learning and Growth.” The commitment to becoming better, both professionally and personally, emerges as a core aspect of resilience in critical care nursing [29]. This commitment to ongoing development not only fortifies their ability to adapt to the unpredictable challenges of the ICU but also underscores the transformative power of resilience in shaping not just the quality of patient care but the very essence of the nursing profession itself. In essence, the perpetual learning journey becomes a conduit for building a resilient foundation that transcends the immediate demands of critical care nursing [30].

Concerning team unity, the collaborative resilience evident in the ICU emphasizes the power of teamwork in mitigating the challenges faced by individual nurses [14]. Expounding upon the subtheme of “Team Unity,” it becomes evident that the unbreakable bond formed within the team is more than just a camaraderie of individuals; it is a strategic fusion of diverse strengths that coalesce into a formidable force. This collaborative strength serves as a cornerstone of resilience, enabling the team to navigate the multifaceted challenges of critical care with a shared sense of purpose [5]. The narratives from the nursing teams unveil a profound interdependence where the burdens of the intense work environment are not borne in isolation but are collectively shouldered, fostering a resilient spirit that strengthens the entire team.

Moreover, the subtheme of “Interprofessional Collaboration” sheds light on the intricate network of professionals contributing to the resilience of the team. The mutual trust, shared responsibility, and unwavering support among healthcare professionals within the ICU create a fertile ground for resilience to flourish [31]. This interprofessional collaboration creates a fertile ground for resilience to flourish, as different disciplines synergize their expertise to address the complex needs of critically ill patients. The seamless integration of skills and perspectives fosters an environment where challenges are met with a comprehensive approach, bolstered by a collective commitment to optimal patient outcomes [32].

Regarding emotional balance, the delicate balance between compassion and clinical precision emerged as a central aspect of resilience. Nurses described their role as emotionally demanding, requiring them to find equilibrium between empathetic patient care and maintaining a professional demeanor [33]. This aligns with the subtheme of “Compassion and Clinical Precision,” emphasizing that resilience is not only about technical proficiency but also about navigating the emotional complexities of critical care with strength and grace.

The emotional demands of witnessing human suffering, coupled with the pressure to deliver precise and life-saving interventions, create a unique tension that resilient nurses transform into strength. This delicate balance is not a static achievement but a continuous process that requires self-awareness, coping strategies, and a supportive environment. Thus, the subtheme highlights that emotional resilience is an integral component of overall resilience in critical care nursing, emphasizing the nurses' capacity to sustain compassionate care while upholding the clinical precision demanded by their profession [1, 34].

The subtheme of “Coping with Challenges” further highlights the emotional toll nurses face. Resilience, in this context, becomes an active coping mechanism – a conscious effort to manage emotional stressors and find inner strength to persevere through difficult situations. Building upon the exploration of resilience as an active coping mechanism, it is crucial to delve into the collaborative unity that emerged as a pivotal component in the coping strategies of critical care nurses [8, 35].

Team-based coping mechanisms, such as regular interdisciplinary meetings and peer support initiatives, not only facilitated the exchange of coping strategies but also created a sense of belonging and shared responsibility. This collaborative approach not only contributed to the resilience of individual nurses but also fostered a resilient culture within the critical care unit, ultimately enhancing the overall capacity of the nursing team to adapt and thrive in challenging circumstances [36, 37].

The resilience of critical care nurses is integral to ensuring positive patient outcomes, including safety, access to care, and satisfaction. Resilient nurses are better equipped to navigate high-stress environments, reducing the likelihood of medical errors and fostering safer patient care practices [35]. Their ability to adapt to evolving situations also ensures that patients receive timely interventions, improving access to essential healthcare services in critical moments. Furthermore, resilient nurses demonstrate an enhanced capacity for empathetic communication, which has been linked to improved patient satisfaction and trust in the care provided [38].

Concerning self-care and well-being, the discussion on resilience in critical care nursing would be incomplete without acknowledging the nurses' recognition of their own vulnerabilities. The subtheme of “Acknowledging Vulnerability” highlights the importance of self-awareness and the acknowledgment that embracing vulnerability is not a sign of weakness but a testament to their humanity. The acknowledgment of vulnerability serves as a catalyst for proactive self-care practices. Nurses in our study described how acknowledging their own emotional fragility prompted a commitment to prioritize self-care without guilt or hesitation. This self-awareness led to the development of personalized coping strategies, ranging from engaging in creative outlets to seeking professional psychological support when needed [39, 40].

“Prioritizing Self-Care,” as another subtheme, is positioned as a professional responsibility rather than a luxury. The study highlights the necessity of intentional steps to nurture well-being, recognizing that sustaining resilience requires proactive self-care practices. Moreover, "Prioritizing Self-Care" extends beyond routine practices; it encapsulates a paradigm where well-being is considered a dynamic and evolving process. Nurses actively engage in reflective practices to assess their unique stressors and adapt their self-care strategies accordingly [41, 42].

Regarding reflection on experiences, the ICU becomes a space for resilience to flourish. Nurses actively engage in “Celebrating Victories,” no matter how small, reflecting on successes as guiding compass points toward hope and renewed strength. This subtheme sheds light on the significance of fostering a positive mindset within the critical care environment. The acknowledgment and celebration of victories, whether minor milestones in patient recovery or successful collaborative interventions, contribute to a culture of positivity and hope [43]. Our study reveals that these celebrations are not mere rituals but intentional acts of self-reflection and appreciation for the impact nurses make on patient outcomes.

Additionally, the subtheme of “Learning from Setbacks” highlights the resilience-building aspects of turning setbacks into opportunities for reflection and improvement. Within the dynamic and often unpredictable environment of critical care nursing, setbacks are inevitable, and our study reveals that nurses approach these challenges with a proactive and adaptive mindset. Instead of viewing setbacks as insurmountable obstacles, participants described a collective commitment to extracting valuable lessons from difficult situations. This resilience-building process involves a continuous cycle of reflection, analysis, and adaptation to enhance individual and collective practices [44, 45].

In the Egyptian context, resilience among critical care nurses is influenced by unique systemic and cultural factors. Limited healthcare resources, staffing shortages, and high patient-to-nurse ratios present additional challenges that demand heightened adaptability and emotional balance [46]. Despite these constraints, participants in this study demonstrated remarkable resilience, reflecting the global patterns of adaptive capacity and collaborative unity while also highlighting specific coping mechanisms tailored to the Egyptian healthcare environment. For instance, reliance on familial and community support networks emerged as a significant factor in sustaining resilience, a finding consistent with literature on the role of social structures in Middle Eastern cultures [47]. These insights align with global findings while emphasizing the importance of localized interventions to enhance resilience.

The findings of this study align with existing literature emphasizing the importance of resilience in critical care nursing globally. Previous research highlights that resilient nurses are better positioned to mitigate burnout, maintain high-quality care, and improve patient outcomes [13, 47]. However, this study adds nuance by exploring resilience through the lens of the Egyptian healthcare context, where systemic challenges and cultural factors uniquely shape resilience. While similarities exist with global findings, such as the significance of teamwork and self-care, this study underscores the distinct strategies employed by nurses in resource-constrained settings, offering valuable contributions to the broader discourse on resilience in critical care nursing [13, 45].

Understanding the dynamic processes of resilience in critical care nursing holds implications for nursing practice, education, and organizational support. Interventions and support programs should be designed to foster continuous adaptation, collaborative unity, emotional balance, and self-care. Moreover, organizations should recognize the collective nature of resilience, promoting a culture that values and supports interprofessional collaboration [1, 45].

The connection between resilience and the work environment is a crucial factor in comprehending its wider implications. Workload, organizational support systems, and the culture of healthcare institutions significantly influence the resilience of critical care nurses. A supportive work environment, characterized by adequate staffing, access to resources, and a culture of recognition and appreciation, amplifies resilience by reducing stress and fostering a sense of belonging [48]. Conversely, unsustainable workloads and lack of organizational support can erode resilience, leading to burnout and reduced quality of care [49]. Addressing these factors is essential for creating environments where resilience can thrive, ultimately improving both nursing experiences and patient care outcomes.

### Strength and limitations of the work

The study demonstrates several strengths. Firstly, it employs a qualitative research design, which is ideal for exploring the intricate and subjective nature of resilience in critical care nursing. The use of qualitative methods allows for a comprehensive understanding of participants' experiences, their perspectives on resilience, and the coping strategies they employ to overcome challenges. Additionally, the study utilizes purposeful sampling to select participants who have demonstrated resilience in their professional roles. This ensures that the data collected accurately reflects the experiences of nurses who have successfully navigated the demands of critical care nursing. Member checking was implemented to enhance the credibility and confirmability of these findings, ensuring that participants validated the emerging themes. To further strengthen the study’s rigor, an audit trail, and peer debriefing with an external qualitative research expert were employed, enhancing transparency and minimizing bias.

Limitations of the study include focusing on nurses with a track record of resilience may introduce self-selection bias and exclude perspectives from nurses with different experiences. The study does not explore specific components of resilience related to outcomes such as job satisfaction, burnout mitigation, or retention rates. The qualitative research design limits the ability to quantify resilience objectively. Potential researcher bias may influence the interpretation of findings, and the lack of longitudinal data prevents capturing the evolution of resilience over time and its influencing factors.

### Recommendations for further research

To further enhance the study, future research could consider longitudinal research designs can be employed to investigate how resilience evolves and fluctuates over time within individuals and across different career stages. Utilizing mixed-methods approaches can provide a comprehensive understanding of the factors that influence resilience and its impact on patient outcomes by combining qualitative and quantitative data.

Additionally, comparative studies can be conducted to examine resilience differences among various healthcare professionals in critical care settings. Lastly, investigating the influence of organizational factors, such as leadership styles, work environment, and available resources, on fostering resilience in critical care nurses would contribute to a deeper understanding of this topic.

### Implications for policy and practice

Develop specialized training programs for critical care nurses based on identified resilience dimensions. These programs should target continuous adaptation, collaborative unity, and emotional balance, providing practical tools to navigate the challenges unique to intensive care settings.

Policymakers and employers should prioritize resilience-building initiatives, such as structured support programs, mental health resources, and resilience training. These measures can enhance job satisfaction, mitigate burnout, and improve recruitment and retention, ultimately fostering a resilient workforce and better patient care outcomes.

## Conclusion

In conclusion, this qualitative inquiry has provided a nuanced understanding of resilience in critical care nursing, highlighting the multifaceted and interconnected aspects that contribute to the dynamic processes within the ICU. The findings of this study contribute to the growing body of knowledge on nursing resilience and offer a foundation for further research and the development of targeted interventions to enhance the well-being of critical care nurses.

## Supplementary Information

Supplementary Material 1.

Supplementary Material 2.

## Data Availability

No datasets were generated or analysed during the current study.
