# Supplementary material for: Exploring resilience in critical care nursing: a qualitative inquiry into continuous adaptation, collaborative unity, and emotional balance
Source: BMC Nurs. 2025 Mar 3;24:238. doi: 10.1186/s12912-025-02844-0 (PMC11877925; doi:10.1186/s12912-025-02844-0)
Supplement: Supplementary file 2 — Supplementary Material 2. [file 12912_2025_2844_MOESM2_ESM.docx]

**Interview Guide for Exploring Resilience in Critical Care Nursing**

**Section 1: Continuous Adaptation**

- Tell me about some of the most common challenges you face in providing care in the ICU.
- How do these challenges differ from those in other nursing settings?
- Describe a specific situation where you had to adapt quickly to a changing environment or patient condition.
- What strategies or skills do you rely on to navigate these unpredictable situations?
- How do you stay up-to-date with the latest developments and best practices in critical care?
- Share an example of a situation where you learned something new from a challenging experience.
- How do you incorporate this learning into your future practice?
- How do you balance the need for innovation with adhering to established protocols in the ICU?

**Section 2: Collaborative Unity**

- Describe how you build trust and collaboration with your colleagues in the ICU.
- What communication strategies do you find most effective in high-pressure situations?
- Share an example of how effective communication within your team positively impacted patient care or team dynamics.
- How do you navigate potential conflicts or disagreements within the team?
- Tell me about your experiences collaborating with other healthcare professionals in the ICU (e.g., doctors, respiratory therapists, social workers).
- Describe a specific instance where interprofessional collaboration significantly impacted patient outcomes or team effectiveness.
- What are some of the challenges you face in fostering effective collaboration across different disciplines?
- How do you advocate for the role of nurses within the interprofessional team?

**Section 3: Emotional Balance**

- Tell me about the most emotionally challenging situations you have encountered in the ICU.
- How do your coping strategies differ when facing stressful situations involving patients, colleagues, or families?
- Describe some self-care practices you use to maintain your emotional well-being.
- How do you seek support from colleagues or other resources when needed?
- Share a story of a patient's recovery or positive outcome that brought you a sense of meaning and purpose in your work.
- How do you maintain your motivation and resilience in the face of difficult situations and losses?
- What brings you joy and satisfaction in your role as a critical care nurse?
